# Complications associated with pre-hospital open thoracostomies: a rapid review

**DOI:** 10.1186/s13049-021-00976-1

**Published:** 2021-12-04

**Authors:** Stian Mohrsen, Niall McMahon, Alasdair Corfield, Sinéad McKee

**Affiliations:** 1 ScotSTAR, Emergency Medical Retrieval Service, 180 Abbotsinch Road, Paisley, PA2 3RY UK; 2 Faculty of Health Sciences and Sport, University of Stirling, Stirling, FK9 4LA Scotland, UK; 3 Department of Nursing, School of Health and Life Sciences, Glasgow Caledonian University, Cowcaddens Road, Glasgow, G4 0BA Scotland, UK

**Keywords:** Emergency medical services, Critical care, Thoracic injuries, Pneumothorax, Thoracostomy, Intraoperative complications

## Abstract

**Background:**

Open thoracostomies have become the standard of care in pre-hospital critical care in patients with chest injuries receiving positive pressure ventilation. The procedure has embedded itself as a rapid method to decompress air or fluid in the chest cavity since its original description in 1995, with a complication rate equal to or better than the out-of-hospital insertion of indwelling pleural catheters. A literature review was performed to explore potential negative implications of open thoracostomies and discuss its role in mechanically ventilated patients without clinical features of pneumothorax.

**Main findings:**

A rapid review of key healthcare databases showed a significant rate of complications associated with pre-hospital open thoracostomies. Of 352 thoracostomies included in the final analysis, 10.6% (n = 38) led to complications of which most were related to operator error or infection (n = 26). Pneumothoraces were missed in 2.2% (n = 8) of all cases.

**Conclusion:**

There is an appreciable complication rate associated with pre-hospital open thoracostomy. Based on a risk/benefit decision for individual patients, it may be appropriate to withhold intervention in the absence of clinical features, but consideration must be given to the environment where the patient will be monitored during care and transfer. Chest ultrasound can be an effective assessment adjunct to rule in pneumothorax, and may have a role in mitigating the rate of missed cases.

**Supplementary Information:**

The online version contains supplementary material available at 10.1186/s13049-021-00976-1.

## Background

In addition to the oesophagus and lymphatic vessels, the thoracic cavity contains several life-sustaining structures including the heart and great vessels, airways, and lungs [1]. Injury to any of these can place a person at immediate threat of severe disability or death, making chest trauma a well-acquainted adversary of emergency pre-hospital care providers. Even though significant chest injuries are associated with adverse outcomes, they can manifest late and have proven difficult to identify on clinical examination [2, 3]. Leech et al. [4] list closed tension pneumothorax (T-PTX) as the most common severe pathology in major chest trauma (1 in 250), a condition where air is increasingly introduced to the pleural space without an ability to escape [1]. This can develop over a matter of minutes or several hours [5], and occurs when a conduit is created by a rupture of lung tissue or an open wound through the chest wall, or a combination of the two. Increasing volume of air in one side of the pleural cavity interferes with pleural adhesion and disrupts the negative-pressure mechanism normal ventilation relies upon. An ever-increasing pleural volume compresses the ipsilateral lung further inhibiting alveolar ventilation area, and rising pressure shifts structures such as the vena cavae contralaterally which reduces cardiac preload and eventually causes circulatory collapse. Significant haemorrhage into the pleural cavity can present a similar mass effect on the lung and mediastinal organs, but less often creates tension and introduces significant intravascular volume depletion as a co-pathology [4, 6].

Immediate mitigation of large T-PTX involves the release of air from the pleural cavity. In its simplest form this is done by inserting an open intravenous cannula through the chest wall and can be performed in the spontaneously breathing patient to alleviate high intrathoracic pressures. This is a safe intervention when applied in the correct circumstances [7] but has also been highlighted as often inadequate by Leigh-Smith & Harris [5] in 2004 and in several studies since [8, 9].

Traditionally, definitive treatment has been tube thoracostomies which involve performing a thoracic incision and placing an indwelling catheter attached to a one-way drainage system, to prevent air from re-entering the chest cavity [10]. More recently, Deakin et al. [11] described an open thoracostomy technique in the positive pressure-ventilated patient where altered ventilation physiology would allow an open conduit between the chest and environment without respiratory failure. Several authors have emphasised the success of this technique [12–14], which now is considered standard treatment for patients in traumatic cardiac arrest, or those who are positive pressure-ventilated with significant pneumo- or haemothorax with ventilatory compromise, where the skill is available [4, 15–17]. A recent systematic review by Sharrock et al. [18] sought to compare the safety and efficacy of needle- and open thoracostomies performed by non-physicians but was unable to establish one as definitively superior.

### Additional file 1 contains an illustration of finger thoracostomies

Simple thoracostomy: (a) The ‘triangle of safety’ is identified by the centre of the axilla, the lateral aspect of musculus latissimus dorsi, and the lateral pectoralis major at the line of the nipple, with the arm fully abducted. (b) A bold incision is made through subcutaneous tissue in the fifth intercostal space at the anterior axillary line. (c) Muscle tissue is then dissected using a blunt instrument e.g., a set of arterial forceps, creating a canal to the parietal pleura which is then breached for access to the pleural cavity. A hiss of air, or ooze of blood or pus may present at this point, depending on underlying pathology. (d) The pleural cavity is explored using a finger, assessing for the position of the lung and any adhesions. The resulting canal is left open to allow air or fluid to escape and prevent compression of the lung. (Illustration by Megan Worsfold).

National guidance only describes the application of pre-hospital open thoracostomies in patients where there is a clinical suspicion of tension-pathology [4, 16]. However experience has shown this practice is implemented by some as a preventative measure, where a pneumothorax (PTX) may or may not be present, to avoid complications e.g., unrecognised T-PTX during transport. Decompression of significant PTX regardless of manifested tension has been promoted in secondary literature [19]. However, this concerns an interfacility setting with the ability to confirm the diagnosis with a chest x-ray, but the authors do not discuss what to do when there is an absence of clinical indicators. The Occult Pneumothoraces in Critical Care (OPTICC) trial [20] suggests it may be appropriate to observe patients receiving positive pressure ventilation (PPV) without overt signs of PTX, but this was an in-hospital study where close monitoring and immediate action was readily available. Pre-hospital critical care is typically delivered with a clinician-to-patient ratio of 2:1 with appropriate monitoring and the ability to decompress a developing tension as it presents. However environmental considerations such as vibration, dim lighting and noise are a few examples of potential barriers to identifying rapid changes in clinical condition [19] in the pre-hospital setting. A literature review was performed to explore potential negative implications of open thoracostomies and discuss its role in mechanically ventilated patients without clinical features of pneumothorax.

## Methods

### Selection criteria

A review across several databases was performed to assess the rate of complications in pre-hospital open thoracostomies. Only patients receiving positive pressure ventilation were included as normal respiratory physiology precludes the need for open thoracostomies in the spontaneously breathing patient [4, 16]. Only cases where at least one thoracostomy was performed pre-hospital was included, as this is the environment of practice the question relates to. The patient with chest injuries is emphasised, but papers discussing PTX from medical causes were also included if pre-hospital thoracostomy was a treatment strategy used.

Open thoracostomy is here defined as a surgical procedure where sharp dissection is used to break the skin of the chest in the 4-5th intercostal space in the anterior axillary line, following which blunt dissection and a finger-sweep creates a conduit between the pleura and the environment but without placing a chest tube. This can include cases where the procedure was performed without a clear clinical need, based on signs or symptoms of significant chest trauma to both sides of the chest or deranged physiology; or where only the side(s) of the chest that were injured were opened. Indwelling chest drains were excluded from the review if placed in the pre-hospital environment, as this procedure carries other risks of complication and would be rare in current pre-hospital practice [10, 21].

Outcomes measured were defined as:

- rate of iatrogenic injury to other structures; bleeding including iatrogenic haemothorax; loss of sensation; chronic neuralgia
- wound infection or empyema
- misplacement
- missed contralateral pathology requiring decompression
- delayed healing defined as time dependent on thoracic drainage, ventilation, or surgical wound care (Table 1).

**Table 1:** Selection criteria

Inclusion	Pre-hospital setting Positive pressure ventilated Single or bilateral thoracostomies Published 2000–2021
Exclusion	Not English language Pre-hospital chest drain Traumatic cardiac arrest Needle thoracostomy only Case–control/qualitative designs

### Search strategy

The PROSPERO and Cochrane Reviews databases were interrogated for any reviews answering the clinical question [22, 23]. No relevant articles were found in Cochrane CENTRAL database for clinical trials. Two related best evidence topics by Pritchard [12, 24] relating to patients with chest injuries and in traumatic cardiac arrest respectively were identified in the BestBETs database [25], but differs from this review as it excluded paediatric patients and included papers where indwelling tubes were inserted pre-hospital and were therefore excluded.

Searchable terms were developed from keywords discovered during scoping searches. Terms were connected using truncation and wildcards, and the “AND” or “OR” Boolean logic-operators: pre*hospital OR out-of-hospital AND; thoracostom*; AND iatrogeni* OR complication* OR infection OR empy?ema OR delay*. Subject headings or MeSH-terms were used across the Cochrane and Ovid interfaces, identified by using the interfaces’ integral heading-browsers. Terms related to age and ventilation status were withheld from the search, and excluded at screening if not agreeing with selection criteria to ensure a sufficient sensitivity and search yield.

Searches were performed across titles and abstracts in Scopus (Elsevier), CINAHL (HDAS), Medline (Ovid SP) and Embase (Ovid SP) on 6th March 2021 (Table 2). Papers were limited to those written in English and published between 2000 to 2021. Results were uploaded to Endnote X9 (3.3, Cite While You Write) referencing software and deduplicated, before manual screening of abstracts and full texts against selection criteria.

**Table 2:** Search strategy

	Terms	Scopus	CINAHL	Medline	Embase
1	Pre*hospital	73 661	18 419	12 588	17 439
2	Out-of-hospital	52 710	6 787	12 624	18 749
3	$ SH/MeSH	N/A	13 537	47 783	48 802
4	OR/1–3	109 233	23 647	61 641	78 986
5	Thoracostom*	6 926	773	2 325	3 262
6	$ SH/MeSH	N/A	1 181	2 997	1 243
7	OR/5–6	N/A	1 712	3 018	3 790
8	Iatrogeni*	207 270	8 983	32 791	44 850
9	Complication*	3 097 082	675 868	947 927	1 390 713
10	Adverse	1 799 456	583 316	534 469	831 783
11	Infection	4 805 210	384 789	1 142 202	1 458 125
12	Sepsis	436 334	31 176	100 668	156 477
13	Empy*ema	30 485	1 605	9 686	11 618
14	Bronchiectas*	36 202	2 132	10 000	16 054
15	Pain	2 147 032	322 318	649 788	969 019
16	$ SH/MeSH	N/A	35 595	103 346	489 506
17	OR/8–16	N/A	1 624 316	3 090 405	4 448 115
18	Delay*	2 801 825	106 906	493 565	670 336
19	Prolong*	1 427 447	56 571	389 541	524 377
20	OR/18 + 19	N/A	159 064	856 233	1 155 597
21	Healing	907 080	73 579	183 322	234 950
22	Recovery	3 017 527	106 412	463 874	614 261
23	OR/21 + 22	N/A	164 306	640 509	839 667
24	AND/20 + 23	517 883	11 516	55 984	79 312
25	$ SH/MeSH	N/A	54 375	92 138	439 343
26	OR/24 + 25	N/A	65 458	147 171	515 468
27	OR/17 + 26	10 240 619	1 656 484	3 180 724	4 756 714
28	AND/4 + 7 + 27	31	31	47	49

^$^ SH/MeSH/Terms = Subject headings/MeSH

For Scopus: N/A

For CINAHL: (3) “Prehospital Care”; (6) “Thoracostomy +”; (16) “Postoperative Hemorrhage” OR “Postoperative Pain” OR “Surgical Wound Infection” OR “Iatrogenic Disease”; (25) “Treatment Duration” OR “Length of Stay”

For Medline: (3) Emergency Medical Services/; (6) Thoracostomy/; (16) Iatrogenic Disease/or Pain, Postoperative/or Postoperative Haemorrhage/or Surgical Wound Infection/; (25) Duration of Therapy/or Length of Stay/

For Embase: (3) Emergency care/; (6) Thoracostomy/; (16) Postoperative complication/OR Postoperative haemorrhage/or Postoperative infection/OR Postoperative inflammation/OR Postoperative pain/OR Surgical infection/OR Surgical injury/; (25) Treatment duration/OR “Length of stay”/

### Additional file 2 contains a PRISMA flowchart demonstrating the search and screen process

### Data collection and analysis

Qualitative analysis of each paper was performed (Additional file 3: Table 1) and rated using the OCEBM Levels of Evidence [26] and GRADE criteria [27]. Data pertaining to complications were extracted, and common types of iatrogenesis or complications were clustered. Incidence of complications was calculated and is presented as counts and percentages of all complications and all thoracostomies. One paper was excluded from quantitative analysis as it did not follow patients beyond handover to the emergency department [13].

## Results

A total of five papers met the selection criteria after full text screening, all pertaining to procedures performed pre-hospital but only four describing outcomes beyond hospital admission [13, 14, 28–30]. The papers included a total of 350 patients receiving 427 thoracostomies (excluding traumatic cardiac arrest), of which 386 (90.4%) were in the pre-hospital environment and 41 (9.6%) were in hospital. Two-hundred-and-twenty-four patients (64%) were followed up past admission with a mortality rate of 28.5% (n = 64).

### Additional file 3 contains a landscape table of included studies with analysis

### Indications and procedure

Open thoracostomies are universally indicated in the presence of a large PTX in patients receiving PPV, as this is associated with an increased risk of developing tension pathology [31, 32]. Massarutti et al. [14] defined a clinical diagnosis of simple PTX as decreased breath sounds, subcutaneous emphysema, serial rib fractures with chest wall instability, flail chest or penetrating chest wounds. Aylwin et al. [30] applied similar criteria but added the presence of a unilateral wheeze to the list of clinical signs and added a wider range of indications including undifferentiated hypotension, or unilateral signs of a PTX in the presence of hypoxia or hypotension. Aylwin et al. [30] used T-PTX as the indication for the procedure defined as hypoxia, hypotension, absent breath sounds and tracheal shift. Conversely, Massarutti et al. [14] defined T-PTX based on the result of the procedure, as determined by an apparent hiss of air and/or rapidly stabilising vital signs following the procedure. Chesters et al. [13], Hannon et al. [28] and Quinn et al. [29] did not elaborate on their clinical indications but included chest injuries presenting a high risk of PTX, or unexplained hypoxia or hypotension in all patients receiving PPV. All papers agreed on finger thoracostomies as appropriate routine measures in traumatic cardiac arrest, and described the procedure uniformly, most referring to the initial description of the technique by Deakin et al. [11] in 1995.

### Complications

Of the 352 procedures followed up past admission, 10.6% (n = 38) were associated with complications and of these 7.3% (n = 26) were caused by procedural error and subsequent injury, infection, or treatment failure, while missed or recurring PTX accounted for 3.4% (n = 12).

Of the 38 complications identified in this review (Table 3), iatrogenic injury including injury to underlying organs, unintended bleeding, induced haemothorax or unnecessarily created thoracostomies was most common (28.9%, n = 11). Failure to decompress underlying PTX despite attempt and misplaced incisions accounted for five (13.1%) and eight cases (21%) respectively, and missed PTX and recurrent tension accounted for eight (21%) and four (10.6%) cases, respectively. Only two cases (5.2%) of post-procedure infection were identified on follow-up, but of note, the use of prophylactic antibiotics is not discussed throughout the papers and can therefore not be assessed reliably. Excluded from these data is the study by Chesters et al. [13] which did not include any follow-up beyond the pre-hospital phase.

**Table 3:** Complications

Complication	No	% (*n* = 38)	% (*n* = 352)
Iatrogenic injury	11	28.9	3.1
Failed procedure	5	13.1	1.4
Misplacement	8	21.0	2.3
Infection	2	5.2	0.5
Recurrent PTX	4	10.5	1.1
Missed PTX	8	21.0	2.2
Total	38	100	10.6

## Discussion

Pre-hospital open thoracostomies have shown to be effective at relieving T-PTX and retain patency, avoiding the time-consumption and complications associated with inserting a drainage tube [33]. Findings from this review support this with only 1.1% of PTX re-tensioning post-procedure, but there is an apparent paucity of studies assessing patient-focused outcomes from thoracostomies with only three of the five identified papers attempting follow-up beyond pre-hospital or emergency department care [14, 28, 30].

Careful balancing of benefit and harm, in line with the core principles of biomedical ethics [33] is required with any invasive procedure. Open thoracostomies are not benign with an overall procedural complication rate of 7.4% (excluding missed and recurrent PTX) and are associated with other complications such as long-term pain and cosmetic implications [10, 34], although these were not discussed in the papers in this review.

Finger thoracostomies in trauma are appropriate in circumstances where the patient has suffered chest injuries and is in cardiac arrest [15, 17]. It is also an established intervention in tension pneumothorax [15, 16], but the criteria for diagnosis are inconsistent between the papers in this review [13, 14, 28–30]. Unilateral chest pathology following trauma with features of reduced or absent air entry, with persistent or worsening hypoxia despite other measures, and/or features of shock or high ventilator airway pressures, should prompt consideration of a tension pathology requiring decompression [9, 16, 31]. However, there are many other potential causes for hypoxia, shock, and high airway pressures in the positive pressure ventilated trauma patient. Therefore, the decision to perform thoracostomy could be helped by using a checklist to ensure other less invasive causes are ruled out, before committing the patient to a surgical procedure [35].

Pre-hospital practice has evolved tremendously since Deakin et al. [11] first described the open thoracostomy method with the recent introduction of point-of-care ultrasound (POCUS) [36]. The technique is increasingly popular and advocated as an adjunctive decision-making tool in chest trauma [15]. POCUS has demonstrated high positive and negative predictive values, but due to significant inter-rater differences it is currently regarded too unreliable in completely ruling out pathology [37, 38]. Chest wall surgical emphysema in particular significantly reduces the utility of POCUS. That said, services that can ensure adequate training and competence should consider incorporating POCUS assessment into their guidelines. Further research on the utility of POCUS in pre-hospital chest injury management may help define those patient groups most likely to benefit from pre-hospital finger throactostomy.

## Limitations

This review has several limitations. All the included studies have used purposive sampling introducing potential selection bias, in this case patients requiring pre-hospital thoracostomies. This patient group typically suffers other major injuries associated with high morbidity and mortality and could confound outcomes, which cannot be adjusted for effectively with retrospective study designs and no comparison groups. Sample sizes are small and heterogenous; additionally, several of the papers demonstrated a high loss to follow-up with a mean of 71.4% (n = 64/224) of patients followed up to survival [14, 28–30]. This attrition bias could significantly affect the outcomes observed in the sample population hiding procedural complications as contributing to mortality in these patients. The retrospective nature of three of the studies [13, 28, 29] raises the possibility of reporting bias from inadequate or inaccurate notetaking. Conversely, prospective studies could encourage clinicians to create notes portraying more favourable outcomes than they otherwise would, knowing their practice is being assessed. Aylwin et al. [30] and Quinn et al. [29] included thoracostomies performed in the emergency department in their data which reduces the generalisability of these results to pre-hospital practice. The study by Hannon et al. [28] also included follow-up data on three cases who initially were in cardiac arrest which is not directly applicable to the selection criteria for this review.

## Conclusion

Pre-hospital thoracostomies are associated with a 10.6% complication rate based on the evidence identified in this review, most of which are due to operator error as opposed to unresolved or missed pathology. An open thoracostomy technique is likely to be as safe or safer than tube thoracostomies and remains the preferred option unless a tube is indicated for other reasons. Occult pneumothoraces can develop tension with subsequent shock or cardiac arrest, but it may be appropriate to withhold intervention in the absence of clinical features depending on the situation rather than ‘empirical’ thoracostomy. Clinicians should consider the environment where the patient will be monitored during care and transfer, and chest ultrasound can be used as an adjunct to assessment. Positive findings of pneumothorax on ultrasound may support a decision to decompress, but a normal ultrasound cannot exclude pathology and continuous patient monitoring remains pertinent. Existing evidence is too weak to establish definitive data on complications following pre-hospital thoracostomies, but this could be improved with prospective observational research with adequate follow-up beyond hospital admission.

## Supplementary Information

**Additional file 1. Figure 1**: Illustration of an open thoracostomy.

**Additional file 2. Figure 2**: PRISMA flowchart of the search and screen process.

**Additional file 3. Table 1**: Landscape table of included studies with summary of analysis.

## Data Availability

All data generated or analysed during this study are included in this published article through (1) tables included in the paper, and (2) the published articles included in the review.

## Abbreviations

POCUS: Point-of-care ultrasound
PPV: Positive pressure ventilation
PTX: Pneumothorax
T-PTX: Tension pneumothorax
