# Supplementary material for: Complications associated with pre-hospital open thoracostomies: a rapid review
Source: Scand J Trauma Resusc Emerg Med. 2021 Dec 4;29:166. doi: 10.1186/s13049-021-00976-1 (PMC8643006; doi:10.1186/s13049-021-00976-1)
Supplement: Supplementary file 1 — Additional file 1. Figure 1: Illustration of an open thoracostomy. [file 13049_2021_976_MOESM1_ESM.pptx]

## Slide 1
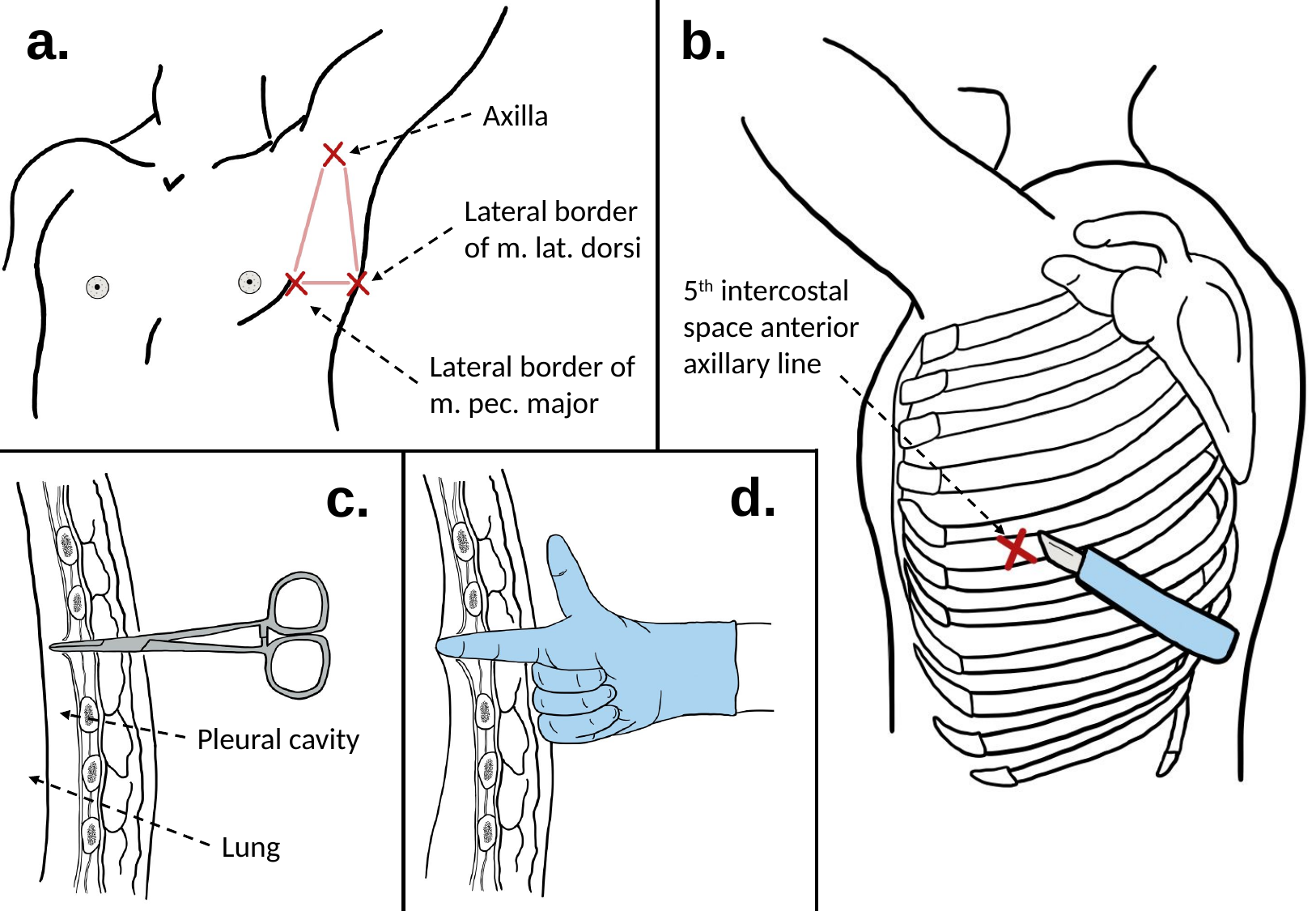

a.
b.
Axilla
Lateral border of m. lat. dorsi
5th intercostal space anterior axillary line
Lateral border of m. pec. major
c.
d.
c.
Pleural cavity
Lung
