# Supplementary material for: Complications associated with pre-hospital open thoracostomies: a rapid review
Source: Scand J Trauma Resusc Emerg Med. 2021 Dec 4;29:166. doi: 10.1186/s13049-021-00976-1 (PMC8643006; doi:10.1186/s13049-021-00976-1)
Supplement: Supplementary file 2 — Additional file 2. Figure 2: PRISMA flowchart of the search and screen process. [file 13049_2021_976_MOESM2_ESM.docx]

Full-text articles excluded, with reasons
(n = 10)

Language (n = 1)

Study design (n = 3)

Outcomes (n = 4)

Cardiac arrest (n = 2)

Studies included in quantitative synthesis
(n = 4)

Records identified through database searching
(n = 158)

Studies included in qualitative synthesis
(n = 5)

Full-text articles assessed for eligibility
(n = 15)

Records excluded
(n = 26)

Records screened
(n = 41)

Records after duplicates removed
(n = 41)

Additional records identified through other sources
(n = 5)

## Identification

## Eligibility

## Included

## Screening
