# Supplementary material for: Complications associated with pre-hospital open thoracostomies: a rapid review
Source: Scand J Trauma Resusc Emerg Med. 2021 Dec 4;29:166. doi: 10.1186/s13049-021-00976-1 (PMC8643006; doi:10.1186/s13049-021-00976-1)
Supplement: Supplementary file 3 — Additional file 3. Table 1: Landscape table of included studies with summary of analysis. [file 13049_2021_976_MOESM3_ESM.docx]

**Table 1: Results**

| Author / Location | Aims / Methods | Sample | Results | Limitations | OCEBM / GRADE |
| --- | --- | --- | --- | --- | --- |
| Massarutti, D. *et al*  2006  Italy | Prospective cohort study evaluating safety and effectiveness of open thoracostomies in traumatic chest injuries, in HEMS-service attached to regional trauma centre. | 55 anaesthetised and intubated patients with decreased breath sounds, subcutaneous emphysema, serial rib fractures with chest wall instability, flail chest or penetrating chest wounds. Patients in cardiac arrest excluded. | Defined tension pneumothorax as apparent hiss of air and/or rapidly stabilising vital signs following the procedure.  Total thoracostomies n=59: unilateral n=51 / bilateral n=4. Survivors n=40 / died in hospital n=15.  No iatrogenic bleeding / complications.  Complications: unnecessary thoracostomy n=2; missed PTX n=6. | Small purposive sample. High loss to follow-up. No comparison group. | 3 + |
| Aylwin, C.J. *et al*  2008  UK | Prospective cohort study assessing indications for pre-hospital thoracostomy and identifying complications associated with pre-hospital and in-hospital pleural drainage procedures over a seven-month period. | 52 patients receiving thoracostomy pre-hospital (n=35) or in ED (n=17). | Tension pneumothorax defined as decreased breath sounds, unilateral wheeze, subcutaneous emphysema, serial rib fractures with chest wall instability, flail chest or penetrating chest wounds, with  hypoxia, hypotension, absent breath sounds and tracheal shift.  Total number of thoracostomies n=91: pre-hospital n=65 / ED n=26.  29% (n=19) pre-hospital thoracostomies had no clear indication.  14% (n=13) complication rate in pre-hospital thoracostomies.  Complications: empyema (n=1); iatrogenic haemorrhage (n=3); failed procedure (n=5); iatrogenic injury (n=1); unnecessary procedure (n=3); misplaced (n=3); re-tensioned (n=4).  9 deceased (unrelated to the procedure). | Small sample. Significant loss to follow-up. No comparison group. | 3 + |
| Chesters, A. *et al*  2015  UK | Case series from case-notes identified in pre-hospital service database describing the type and number of patients who received pre-hospital thoracostomy over a four-year period, seeking to identify any documented pre-hospital complications and observe the difference in patient oxygen saturations before and after the intervention. | 126 patient case records containing the phrase “thoracostomy” in a pre-hospital critical care service. | No clear definition of tension pneumothorax.  Total number of thoracostomies n=236: unilateral n=16 (6.8%) / bilateral n=220 (93.2%). Not in cardiac arrest n=75 (31.7%).  No recorded complications. | No follow-up beyond pre-hospital phase. Vulnerable to reporting bias. Single-centre, small purposive sample. | 4 + |
| Hannon, L. e*t al*  2020  Australia | Retrospective cohort study using case notes to determine the number of finger thoracostomies performed by flight paramedics, the incidence of radiological tension pneumothorax on arrival to hospital and the incidence of complications. | 103 patients undergoing pre-hospital thoracostomy. Adult and paediatric. | No clear definition of tension pneumothorax.  Total number of thoracostomies n=179; bilateral n=152 (85%) / unilateral n=27 (15%).  63 cases (35.2%) survived to follow-up.  Complications (n=3, 4.8%): infection n=1; iatrogenic bleeding n=1; iatrogenic injury n=1. | Includes a small number (n=3) of patients surviving cardiac arrest. Big loss to follow-up. No comparison group. Reporting bias. | 3 + |
| Quinn, N. e*t al*  2020  Australia | Case series from case-note review reporting the early experiences of thoracostomy in severely injured children in a trauma system (pre-hospital and ED) | 14 paediatric patients receiving thoracostomy pre-hospital n=8 (57.1%) / ED n=6 (42.9%). | No clear definition of tension pneumothorax.  Total thoracostomies n=23; bilateral n=18 (78.2%) / unilateral n=5 (21.8%).  2 patients (8.7%) required conversion to bilateral thoracostomy in ED.  No reported complications in either phase of care. | Very small sample. No comparison group. Paediatric population only. | 4 + |

ED = Emergency department; HEMS = Helicopter Emergency Medical Service; PTX = Pneumothorax
